# The psychological reactions after witnessing a killing in public in a Danish high school

**DOI:** 10.3402/ejpt.v4i0.19826

**Published:** 2013-01-09

**Authors:** Ask Elklit, Sessel Kurdahl

**Affiliations:** 1 National Centre for Psychotraumatology, University of Southern Denmark, Odense, Denmark; 2 Ringkøbing-Skjern Municipality, Ringkøbing, Denmark

**Keywords:** witnessing school killing, PTSD, social support, risk factors

## Abstract

**Background:**

School killings attract immense media and public attention but psychological studies surrounding these events are rare.

**Objective:**

To examine the prevalence of posttraumatic stress disorder (PTSD) and possible risk factors of PTSD in 320 Danish high school students (mean age 18 years) 7 months after witnessing a young man killing his former girlfriend in front of a large audience.

**Method:**

The students answered the Harvard Trauma Questionnaire (HTQ), the Crisis Support Scale (CSS), and the Trauma Symptom Checklist (TSC).

**Results:**

Prevalence of PTSD 7 months after the incident was 9.5%. Furthermore, 25% had PTSD at a subclinical level. Intimacy with the deceased girl; feeling fear, helplessness, or horror during the killing; lack of expressive ability; feeling let down by others; negative affectivity; and dissociation predicted 78% of the variance of the HTQ total scores.

**Conclusion:**

It is possible to identify students who are most likely to suffer from PTSD. This knowledge could be used to intervene early on to reduce adversities.

Although they are less frequent than other types of trauma, school shootings and killings result in massive public attention because it is easy to imagine yourself or your children as targets in daily environments. Nevertheless, rather few studies have been performed on this phenomenon and its psychological consequences for the witnesses. Some studies have been carried out on witnessed killings in the workplace or in public, mainly with adults (Creamer, 1989; Elklit, 1994; Johnson, North, & Smith, 2002; North, Smith, McCool, & Shea, 1989; North, Smith, & Spitznagel, 1994, 1997; North, Spitznagel, & Smith, 2001) but also with children (Pynoos, Frederick, Nader, & Arroyo, 1987; Schwarz & Kowalski, 1991). All of the studies pointed to symptoms of posttraumatic stress disorder (PTSD) as a possible consequence of witnessing killings or murders in public. Also, being a witness to a killing had a significant impact on most people, but the degree of trauma was influenced by individual factors.

Most importantly, the effects of witnessing a killing at school and possible risk factors for development of PTSD have to our knowledge never been studied within a high school population. Although there is some research on the effect of disasters on adolescents (e.g., Dogan, 2011; Ghazali, Elklit, Yaman, & Ahmad, 2012) and we know quite a lot about the negative effects of community violence (i.e., witnessing of killing, robbing, threats with weapons, etc.) on adolescents’ development, studies of community violence are normally conducted in high-risk adolescent populations, who have experienced multiple traumatic events, and where the possible risk factors for the development of psychopathology are confounded, for example, living in high-risk neighborhoods, having low socioeconomic status, living with parents involved in criminal activity, and possible substance abuse (Foy, Ritchie, & Conway, 2012; Stein, Jaycox, Kataoka, Rhodes, & Vestal, 2003). Almost nothing is known about the effects of witnessing of a single incident of high school killing in typical (non-high-risk) adolescent populations. The goal of the current study was to identify demographic, peri-traumatic, and psychosocial factors that influenced the degree to which the experience of the high school killing affected psychological adjustment (expressed by severity of PTSD symptoms) in adolescent students, 7 months after the incident. Well-established risk and protective factors from the PTSD literature were chosen for investigation in the present study.

*Gender* plays an important role in the risk of traumatization. Two meta-analyses (Brewin, Andrews, & Valentine, 2000; Tolin & Foa, 2006) concluded that women are more likely than men to meet the criteria for PTSD after a traumatic event, even though they are less likely to experience potentially traumatic events (see also Olff, Langeland, Draijer, & Gersons, 2007). The twofold risk for PTSD among women could not be attributed solely to a higher frequency of specific, highly pathogenic potentially traumatic events such as sexual assault, but seemed to exist across trauma categories. In relation to witnessing public killings, North et al. (1994, 1997, 2001) reported a corresponding difference with 20 and 36% of the men and women, respectively, having PTSD 1 month after the incident. The participants in their study had all witnessed a gunman shooting in a cafeteria, but early in the gunfire it became obvious that the shooter favored killing females. Therefore, it is unclear whether this gender-related risk affected the difference in the level of traumatization in male and female witnesses in this particular study.

*The level of intimacy with the victim* has been shown to influence the witnesses’ PTSD severity. Dyregrov, Frykholm, Lilled, Broberg and Holmberg (2003) investigated 563 adolescents’ reactions (aged 13–19 years; mean age 15.4) 7 months after a discotheque fire in Sweden that killed 63 young people. The levels of the participants’ traumatic reaction increased with increased intimacy, that is, knowing the deceased personally. The same results have been found in other studies of child, adolescent, and young adult witnesses of mass killings, natural disasters, and peers to adolescent suicide victims (Melhem et al., 2004; Parslow, Jorm, & Christensen, 2006; Pynoos et al., 1987). This indicates that particular attention should be given to those who perceived themselves to be psychologically close to the deceased.

*The A2 criterion in the PTSD diagnosis:* The exposure criterion in the PTSD diagnosis in DSM-IV has two elements: Criterion A1 specifies that the person experienced, witnessed, or was confronted with events involving actual or threatened death, physical injury, or other threats to the physical integrity of the self or others; criterion A2 requires that the initial response to the event involves fear, helplessness, or horror (American Psychiatric Association, 1994). Criterion A2 is known to be a very good predictor of subsequent PTSD (e.g., Brewin, Andrews, & Rose, 2000; Parslow et al., 2006).

In general, it appears that *social support* from family and friends has a positive influence on the ability to cope with a trauma (Olff, 2012). Out of 14 separate risk factors for PTSD investigated in a meta-analysis (Brewin et al., 2000), social support was the strongest predictor accounting for 40% of variance in PTSD severity. When studies investigated both positive and negative support elements, a negative social environment was a better indicator of PTSD symptomatology than lack of positive support (Brewin & Holmes, 2003). The perception of social support is also important in the prediction of PTSD. In a study of 150 undergraduate students aged 17–22 years (mean age 19.3) who reported having experienced different types of trauma, Haden, Scarpa, Jones, and Ollendick (2007) investigated the moderating effect of perceived social support on PTSD and perceived injury. The researchers concluded that the concept of support, perceiving it or seeking it out, was one of the most powerful variables essential for minimizing the severity of PTSD in young adults.

*Negative affectivity* refers to a mood dimension and reflects a general tendency to react to and have a negative perspective on the surrounding world and oneself (Krog & Duel, 2003; Watson & Clark, 1984). Negative affectivity can either be seen as a state or as a personality factor similar to neuroticism (ibid.). Significant associations between PTSD and negative affectivity are often found in different trauma populations (e.g., Bennett, Owen, Koutsakis, & Bisson, 2002; Feldner, Lewis, Leen-Feldner, Schnurr, & Zvolensky, 2006; Monson, Price, Rodriguez, Ripley, & Warner, 2004; Shapinsky, Rapport, Henderson, & Axelrod, 2005).

*Dissociation* may be understood as responses occurring in the course of a traumatic event, which cause a disruption in the usually integrated functions of perception of the environment, memory, consciousness, or identity (American Psychiatric Association, 1994; Bryant, 2007).

Dissociative responses that occur at the time of trauma (peritraumatic dissociation) have been described as the largest known risk factor for subsequent PTSD (Ozer, Best, Lipsey, & Weiss, 2003). However, Briere, Scott, and Weathers (2005) showed that the less-investigated, trauma-specific dissociation, that occurs during or after an event and continues until the time of assessment (persistent dissociation), when taken into account, pushes aside the effect of peritraumatic dissociation on PTSD. Trauma-related persistent dissociation is also found to be a substantial predictor of PTSD in other studies (Ehlers & Clark, 2000; Halligan, Michael, Clark, & Ehlers, 2003; Murray, Ehlers, & Mayou, 2002).

In spite of the fact that this is a retrospective study (conducted 7 months after the incident), its importance is highlighted by the lack of other recorded studies of this specific trauma-type in adolescents. Given the relatively rare occurrence of public high school killings, and even more rare scientific investigation of them, identification of possible risk factors of PTSD in this particular incident can be of value in guiding future screening programs to prevent chronification of symptoms in the adolescent victims.

## The incident

The killing at the Hasseris High School in Aalborg, Denmark, occurred on Friday, March 3, 2006, just when the annual Shrovetide party was getting started. A female student ran through the front door into the open wardrobe area closely followed by her former boyfriend with whom she had broken up with 2 days earlier. He held a bloodstained knife in his hand and she was screaming. After overpowering her in the middle of the student crowd, he stabbed her several times in the chest. Most of the students thought that what they saw was some kind of theater performance and did not interfere. They slowly became aware of the severity, when the stabber fled and the police and an ambulance arrived. Almost 200 party participants were detained inside the assembly hall and a classroom for 3 hours until the police had found the killer, who had hung himself in a shed near his home. In the meantime, it was difficult for the students to find out what had happened, and students and family members outside the high school had problems contacting their friends and relatives inside the school because of an overloaded mobile telephone network. The next day, the headmaster and a crisis psychologist held an information meeting for all of the students, relatives, and teachers about the incident and three local psychologists offered counseling for those in need during the following days.

## Method

### Participants

All 415 students, who were attending the high school at the time of the incident, were asked to participate in the study. A total of 320 (77%) returned the questionnaire. The respondents were aged 16–20 years, with a mean age of 17.99 years (*SD*=1.05). Of these, 62.2% were female (199 persons). The parents of the students were well educated: 36% of the fathers and 26% of the mothers had a university degree, and only 11% of the fathers and 9% of the mothers had ended their education after 9 years in public school. Just five (2%) of the students did not have Danish citizenship and 19 (6%) were born outside Denmark. The majority of the students (220=69%) lived with both of their parents, and 76 (24%) lived with only one parent. One fourth (82 persons) of the students had experienced at least one potential traumatic event in their life and 45% (128 persons) had experienced, as a minimum, one important life event during the past year.

### Procedure

The study took place 7 months after the incident. The questionnaires were handed out to the students in second and third grade at the high school and sent to the parents’ addresses of those students who graduated in June (Danish high schools, “gymnasiums”, have three grade levels).

### Instruments

The questionnaire included questions concerning demography and peri-traumatic factors (e.g., spatial proximity to the killing, psychological closeness to the victim) along with the following instruments:

The Harvard Trauma Questionnaire—Part IV (HTQ; Mollica, Caspi-Yavin, Bollini, & Truong, 1992) consists of 16 items scored on a 4-point Likert scale. It measures the intensity of the three core symptom groups (intrusion, avoidance, and arousal) of PTSD. The Danish version of the HTQ has good psychometric properties, comparable to the original (Bach, 2003). The alpha values for three scales in the present study were 0.83 (intrusion), 0.70 (avoidance), and 0.82 (arousal); the total scale with alpha was 0.93. To qualify for the full diagnosis, the appropriate number of core symptoms should be ≥3 (“often”).

The Crisis Support Scale (CSS) was used for rating the experience of perceived social support after a traumatic event through seven items (Joseph, Andrews, Williams, & Yule, 1992). The items include: (1) perceived availability of someone to listen; (2) contact with people in a similar situation; (3) the ability to express oneself; (4) received sympathy and support; (5) practical support; (6) the experience of being let down; and (7) general satisfaction with social support. The items are rated on a 7-point Likert scale, ranging from “never” to “always.” The CSS has been used in several disaster studies—it has good internal consistency as well as good discriminatory power. Elklit, Pedersen, and Jind (2001) have confirmed the psychometrical reliability and validity of the CSS in Danish. Alpha for the total CSS score in the present study was 0.73.

The Trauma Symptom Checklist (TSC) (Briere & Runtz, 1989) measures the occurrence of psychological symptoms associated with trauma. In the present study, a Danish version with 26 items (TSC-26) was used (Krog & Duel, 2003). The Danish TSC has good psychometric qualities and is a valid instrument measuring the effects of traumatization (ibid.). The TSC covers three dimensions and is scored on a 4-point Likert scale. The alpha values in the present study were α=0.83 (negative affectivity; 11 items), α=0.83 (somatization; 10 items), and α=0.70 (dissociation; 5 items); total TSC α=0.91.

### Data analyses

Rates are reported as raw numbers and percentages. Means are reported with standard deviations. The following preliminary tests were conducted to test the association between the presumed risk-factors and symptoms of PTSD: χ^2^-analyses were performed to test associations between dichotomous variables. Correlations were estimated with Pearson's correlation coefficient. ANOVA analyses were used to investigate associations between dichotomous and scale variables and to assess effect sizes. Variables that had significant associations with PTSD symptoms were subsequently entered in a hierarchical regression analysis.

## Results

### Exposure to the killing

Sixty-four persons (19%) were eyewitnesses to the crime; 71 (22%) were in the assembly hall next to the wardrobe area; 48 (15%) were elsewhere in the high school; 18 (6%) were outside, but went inside; 38 (12%) were outside and never got in; 85 (27%) did not take part in the party; one person did not answer. The students outside could see the dead body as the front door was made of glass. During the killing, 189 (59%) reported feeling helpless and powerless, 161 (50%) felt horror and 8 (3%) thought they were going to die. These feelings from now on referred to as “A2”.

### Intimacy with the deceased

Three percent (9 persons) knew the murdered female student “very well” and 6% (18 persons) knew her “well”. Nine percent (28 persons) knew her “some”, 21% (68 persons) “a little”, and 61% (195 persons) did not know her at all. Two persons did not answer this question.

### Posttrauma social support

The CSS total score had an average of 40 (*SD*=5.7) indicating that the students in general experienced good social support. During the following days, there were mourning ceremonies and class talks with counselors. In addition, 35% (112 persons) spoke individually with a psychologist at the high school, 19% (61 persons) with their curriculum counselor, and 10% (32 persons) with the principal. Eight percent (25 persons) were in contact with a crisis psychologist outside the school and 7% (21 persons) with their general practitioner. Six percent (16 persons) indicated that they still felt in need of psychological treatment 7 months after the killing.

### PTSD symptomatology

Seven months after the killing, 28 persons (9.5%) met the three symptom-cluster criteria of a PTSD diagnosis. In addition, 74 persons (25%) met the criteria for two PTSD symptom clusters and for a diagnosis of subclinical PTSD.

### Factors associated with posttraumatic symptomatology

#### Correlations

Correlations were computed between HTQ total scores and all other variables to identify factors associated with posttraumatic symptomatology as assessed by the HTQ. Significant correlations between HTQ total and demographic and peri-traumatic variables included intimacy (*r*=0.28, *p*<0.0005), CSS item 3: lack of expressive ability (*r*=0.37, *p*<0.0005), CSS item 6: feeling let down (*r*=0.49, *p*<0.0005), TSC: negative affectivity (*r*=0.76, *p*<0.0005), and dissociation (*r*=0.69, *p*<0.0005). One-way ANOVA analyses showed a positive relationship between the HTQ total score and female gender (F_(1,278)_=40.88, *p*<0.0005; mean HTQ for males=46.2 (SD 9.6) and for females=57.2 (SD 16.1; η^2^=12.8) and the A2 criterion (F_(3,276)_=44.95, *p*<0.0005; HTQ for no A2=41.6 (SD 10.5) and for A2=55.9 (SD 14.5); η^2^=14.8).

#### Regression analysis

The first step consisted of gender, and the second step of peri-traumatic factors (intimacy and A2), because these three factors were assumed to stay constant across the course of the 7 months since the exposure to the incident. The third step consisted of aspects of social support (CSS item three and CSS item six), and the fourth step of individual differences in reactions to trauma (TSC negative affectivity and dissociation). The final model included six variables, which in all explained 78% of the total HTQ score (Table 1). The contribution of gender was no longer significant in the last step. The variables with the strongest individual contribution to the level of PTSD symptoms were (in order of appearance) negative affectivity, persistent dissociation, and lack of social support (“feeling let down”).

**Table 1:** Hierarchical regression analyses for variables predicting posttraumatic severity in witnesses to a public killing (*n*=269)

Step	Variable	β	F	df	Adj. R^2^	ΔR^2^
1	Gender	0.35***	38.0	1,268	0.12***	
2	Gender	0.21***	51.2	3,266	0.36	0.24***
	Intimacy	0.17**				
	A2	0.45***				
3	Gender	0.15**	62.1	5,264	0.53	0.17***
	Intimacy	0.14**				
	A2	0.39***				
	Lack of expressive ability	−0.26***				
	Feeling let down	0.29***				
4	Gender	0.06	140.2	7,262	0.78	0.25***
	Intimacy	0.08*				
	A2	0.18***				
	Lack of expressive ability	−0.12***				
	Feeling let down	0.21***				
	TSC negative affectivity	0.36***				
	TSC dissociation	0.33***				

* *p*>0.01

** *p*<0.001

*** *p*<0.0005.

## Discussion

Seven months after the incident, 35% of the students were traumatized to at least a subclinical degree. This degree of traumatization existed in spite of the fact that every student in addition to the collective interventions was offered individual sessions with a crisis psychologist closely after the incident (which 1/3 accepted). The Cochrane Collaboration made a comprehensive review (Rose, Bisson, Churchill, & Wessely, 2002) of a single session individual debriefing and found no evidence of it as a useful treatment for the prevention of PTSD after traumatic incidents.

We found that a considerable amount (78%) of the variance in the level of traumatic response could be predicted with six well-known risk factors from the general PTSD literature. First, because of the cross-sectional nature of this study and the delayed measurement of the traumatic effects, some of the significant predictors could be interpreted as a result of the traumatization, rather than risk factors. That is, lack of social support can be seen as a consequence of having developed PTSD symptoms, which in turn hinders social support seeking (Kaniasty & Norris, 2008). Also, at the time of the measurement, the dissociation levels can be seen as a representation of pathogenic traumatic reactions akin to PTSD. This is the most logical explanation because the very definition of PTSD includes some dissociative symptoms. In natural remission from trauma, dissociation symptoms fade away with time (Dalenberg & Carlson, 2012). Persistent dissociation can therefore, in the present population, like in many other traumatized populations (see the introduction), most likely be indicative of a treatment demanding reaction to trauma exposure. This is an important point, because it enables us to identify adolescents in need of more intensive treatment.

Contrary to this, negative affectivity and lack of expressive ability can almost certainly be interpreted as stable individual factors (i.e., personality traits) and therefore possible risk factors that influence the individual reactions to trauma. Difficulties in labeling and communicating emotions are central ingredients of alexithymia. Negative affectivity/neuroticism is related to alexithymia (Wise & Mann, 1994), and the lack of ability to express emotions about the traumatic event might very well be the facet of the overall neurotic reaction pattern. Although no firm conclusions about the causality of the factors can be made because of the cross-sectional design of this study, negative affectivity seems to act as a predisposing factor to more severe traumatic reactions in the present adolescents. This is the most likely interpretation in light of the abundant former research with adults that points to the same associations between trauma and stable traits like negative affectivity.

In light of the retrospective nature of this study, the predictive power of the peri-traumatic factors (A2 criterion, and the intimacy with the victim) might have been overridden by the simultaneous measuring of factors, which at the time of the assessment (7 months after the trauma), are probably indicators of developing pathological reactions to trauma (e.g., dissociation, and lack of social support). This means that the feeling of horror and helplessness during the trauma, and intimate knowledge of the victim, might have had stronger predictive power than dissociation and feelings of being let down, had they been measured more closely to the incident itself. As pointed out in the introduction, the A2 criterion and the intimate knowledge of the victim are also well-known risk factors for the development of PTSD in other traumatized populations. In addition, they are both easily assessed. Therefore, we propose that they be used as the first line of screening in the more immediate aftermath of similar incidents.

Furthermore, the possibility that witnesses in risk of developing PTSD to some degree might have a lack of expressive ability needs to be considered when offering crisis intervention as this might lower the probability of the witnesses seeking help. In Denmark, after a potentially traumatic event such as rape, death of loved ones, physical assault, accidents, and others, crisis intervention is available for the general population (Elklit & Nielsen, 2008). Taking a lack of expressive ability into account, those at risk should then not only be offered help but also be strongly advised to seek help in a proactive way. This is an especially important consideration in relation to adolescent witnesses, whose further development might be jeopardized by persistent traumatic symptoms. Ehlers and Clark (2003) support the need for strategies to identify people who are unlikely to recover on their own. Similarly, the Cochrane review (Rose et al., 2002) concludes that the compulsory debriefing of victims of trauma should cease and the focus of early intervention should instead, like we suggest, be targeted at the risk group. The development of screening instruments and intervention programs is therefore of great importance.

The generalizability of the results of this study is limited by: the cross-sectional design, lack of control group, and the time between incident and assessment, as well as the use of self-reporting. Further research is therefore needed of killings in a school context to investigate the predictors of PTSD in adolescent witnesses to killings and other types of public traumas. Even so, the associations between aspects of trauma and subsequent traumatic reactions, which are known from the adult populations, are being confirmed in adolescent witnesses of public killings in the present study. This provides us with valuable information that can be acted upon, until we have better studies to inform us.

## Conclusion

This study confirms that being a witness to a killing can be a very traumatic experience and identifies six central peri- and posttraumatic variables, which explain a substantial amount of the variation in PTSD. Screening for the variables, that is, intimacy with the deceased, A2 criterion, lack of expressive ability, feeling let down by others, negative affectivity, and dissociation in the witnesses after a similar incident may well be useful to identify those who will develop PTSD and thereby constitute a group on which further psychological treatment should be concentrated.
